# Resilience of health systems in conflict affected governorates of Iraq, 2014–2018

**DOI:** 10.1186/s13031-021-00412-2

**Published:** 2021-10-18

**Authors:** Shaimaa Ibrahim, Sara Al-Dahir, Taha Al Mulla, Faris Lami, S. M. Moazzem Hossain, Abdullah Baqui, Gilbert Burnham

**Affiliations:** 1 UNICEF Iraq Country Office, UN Compound, Baghdad, 10011 Iraq; 2 Xavier University of Louisiana, 1 Drexel Dr, New Orleans, LA 70125 USA; 3 University of Baghdad, Medical City, Baghdad, 00964 Iraq; 4 UNICEF PD, New York, NY 10017 USA; 5 Johns Hopkins Bloomberg School of Public Health, 615 N Wolfe Street, Baltimore, MD 21210 USA

**Keywords:** Iraq, ISIS, Conflict, Primary Health Care

## Abstract

**Objectives:**

The objective of this study was to assess the resilience of health systems in four governorates affected by conflict from 2014 to 2018, and to convey recommendations.

**Methods:**

Health managers from Al Anbar, Ninawa, Salah al-Din, and Kirkuk governorates discussed resilience factors of Primary Health Care services affected by the 2014–2017 ISIS insurgency in focus groups, and general discussions. Additional information was gathered from key informants and a UNICEF health facility survey. Three specific aspects were examined: (1) meeting health needs in the immediate crisis response, (2) adaptation of services, (3) restructuring and recovery measures. Data from a MoH/UNICEF national health facility survey in 2017 were analyzed for functionality.

**Results:**

There were many common themes across the four governorates, with local variations. (1) *Absorption* The shock to the public sector health services by the ISIS invasion caught health services in the four governorates unprepared, with limited abilities to continue to provide services. Private pharmacies and private clinics in some places withstood the initial shock better than the public sector. (2) *Adaptation* After the initial shock, many health facilities adapted by focusing on urgent needs for injury and communicable disease care. In most locations, maternal, neonatal, and child health (MNCH) preventive and promotive PHC services stopped. Ill persons would sometimes consult health workers in their houses at night for security reasons. (3) *Restructuring or transformative activities* In most areas, health services recovery was continuing in 2020. Some heavily damaged facilities are still functioning, but below pre-crisis level. Rebuilding lost community trust in the public sector is proving difficult.

**Conclusion:**

Health services generally had little preparation for and limited resilience to the ISIS influx. Governorates are still restructuring services after the liberation from ISIS in 2017. Disaster planning was identified by all participants as a missing component, as everyone anticipated future similar emergencies.

## Background

**Fig. 1:**
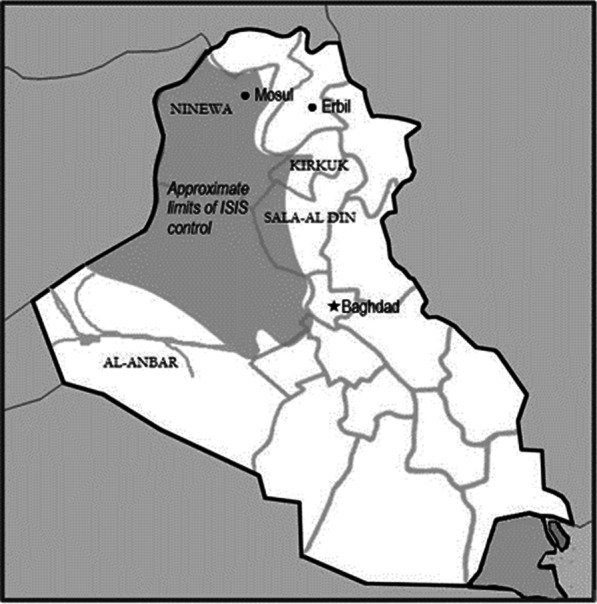
Approximate extent of ISIS control in Iraq, 2015

In January 2014, fighters of the Islamic State of Iraq and Syria (ISIS) seized Ramadi, capital of Iraq’s Al Anbar governorate and shortly after that, Fallujah, Al-Anbar’s principal city [1]. In June 2014, control of Mosul, Iraq’s second city was seized and soon afterwards almost all the Ninewa governorate. During the next months, the invasion of parts of two other governorates, Salah al-Din, and a small part of Kirkuk occurred (Fig. 1). ISIS control in Iraq effectively ended with the recapture of west Mosul by Iraqi government forces June 2017. The conflict displaced nearly 6 million people. In 2021, 1.3 million people remained internally displaced, mainly in the north and west of the country, while 4.7 million have returned to their place of origin [2]. The impact of ISIS on a health system, already weakened by years of conflict and underfunding, was great [3, 4]. Many health facilities had little warning of the impending assault by ISIS, although the jihadists had been active for a period of time in some locations. Many health workers fled, particularly those from minority ethnic and religious groups. Some facilities continued to function under ISIS control, but other facilities were abandoned or sometimes destroyed by military action [5]. In Salah al-Din, 36% of health facilities were destroyed and by 2018, only 30% of hospital beds in Ninewa were functioning [6]. Facilities in these areas often had difficulty obtaining medicines and vaccines, as well as difficulties retaining and paying staff. There is a widespread perception that recovery of civil society post ISIS has stalled in the four governorates, with health services severely impaired [7].

Some health facilities adapted to functioning under these changed circumstances, while others did not. This study was carried out to better understand the resilience of health facilities to provide services in the four conflict-affected governorates.

**Fig. 2:**
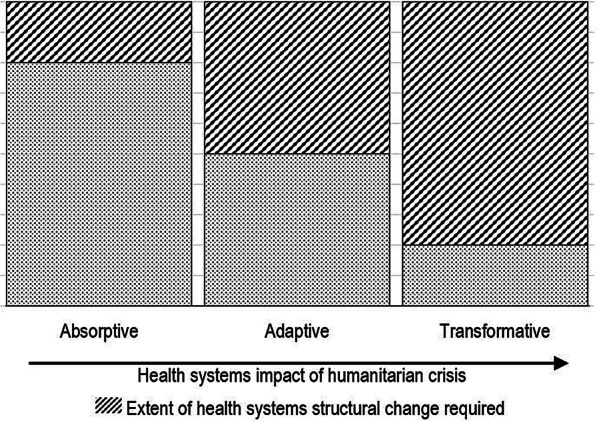
Resilience capacities needed in response to humanitarian crises (after Blanchet et al. [22])

Resilience analysis has become recognized as an important process to assess the capacity of populations exposed to insecurity and conflict. The term resilience is often used in general terms to include governance issues, imbalances of power, the political economy of violence and inequality. These elements of resilience are seen as an important consideration for development programs [8]. Mitchel defined resilience more specifically as the capacity to absorb and recover from shocks whilst positively adapting and transforming services to retain control over service structure and continuity of function in the face of long-term change and uncertainty. The greater the impact of humanitarian crises, the more adaptive and transformative changes will be required to sustain continuity of services (Fig. 2) [9].

The Organization for Economic Co-operation and Development (OECD) developed a systems analysis approach defining resilience according to the various organizational strata as well as the phase of a crisis or degree of instability [10]. Common resilience terminology define three strata. *Absorptive capacity* is the ability of a health system to continue to deliver services in the face of a shock to the system. *Adaptive capacities* are those abilities of the health system to modify or change in order to minimize damage and take advantages of opportunities to meet population health needs. *Transformative capacities* describe the ability to reorganize health services to deliver effective services with new realities. Resilience strata can be defined according to the levels of a system from individual and community through to the executive or ministerial level.

The concept of health systems resilience in disasters became a topic of interest after the 2014 Ebola outbreak in West Africa [11]. Subsequently, resilience analysis has been applied to health systems affected by conflict. A 2019 qualitative analysis examined the capacity of the United Nations Relief and Works Agency (UNRWA) to accommodate the needs of a sudden influx of Palestinian refugees and also the capacity to provide services inside Syria [12, 13]. A data-driven approach was used by Odhiambo et al. [14] to measure health systems resilience in South Sudan. A systems dynamics approach was used by Ager and colleagues to assess health service resilience in Yobe state, Nigeria, in the context of the Boko Haram insurgency [15]. Increasingly, health systems resilience is not seen as an isolated characteristic, but a product of interactions between various complex systems which interact to influence the function of health services.

These findings recognized that a capacity-orientation for health system resilience is important to understanding management of health systems in adverse circumstances. The goal of health systems resilience analysis is to develop the capacities for the health services to face potential instability and shock while continuing to provide services. These are important considerations for Iraq which has endured an almost continuous series of shocks to health systems from 40 years of conflict.

The purpose of this study was to assess the resilience of primary health care (PHC) services in the four governorates which experienced conflict from ISIS between 2014 and2018. The objectives were to pinpoint specific failures of PHC services and identify positive resilience examples with the intent of conveying specific governorate-level recommendations for improving PHC resilience for future crises.

## Methods

During November 2019, two-day meeting was conducted in Erbil for focus group discussions. The 30 participants were divided among four focus groups to discuss the impact of conflict on the population, health care providers and the larger health system. In addition to factors and forces affecting overall PHC services, the specific impact on maternal, neonatal and child health (MNCH) services during the 2014–2017 ISIS conflict was considered. This meeting engaged participants from health directorate management teams, MNCH program managers, and providers working at level of service delivery in the conflict-affected governorates of Al Anbar, Ninewa, Salah al-Din, and Kirkuk. The agenda provided participants an opportunity to reflect on health system resilience of various levels and components, in their own governorates during the ISIS conflict, and to suggest systems changes to improve health system resilience. Four separate focus group discussions were held, each reflecting experiences of each governorate on immediate response, absorption, and recovery phases. The focus groups were, followed by summaries, plenary discussions and queries from participants to the health staff from the various governorates concerning materials in their respective presentations. This helped identify common issues across governorates as well as those particular to a specific location. Discussion of these points were followed up with additional information from key informants working with the central MOH (program managers), and with the analysis of MoH/UNICEF health services data. These additional informants who had also been involved with health services in the ISIS affected areas helped triangulate information from focus groups, adding additional details but not substantially changing opinions or conclusions of the participants.

Participants were given a series of questions to start discussions about health systems resilience from the standpoint of their personal experience and that of their health system responsibilities around the themes and general categories of questions listed below.

1. *Meeting health needs in the immediate response to conflict* In this the emphasis was on the community, public sector PHC centers and health staff, but questions included the private sector and support by the governorate’s Directorate of Health (DoH).
2. *Adaptation and accommodation* This discussion probed how communities and health services adapted to changes brought on by conflict. The capacity to improvise and create alternative approaches during the conflict years was queried.
3. *Restructure and recovery measures post-ISIS* Discussions focused on how community structures emerged post-ISIS. Actions that transformed and re-oriented facility-level services were discussed. A key topic was the preparation of the re-oriented services for future shocks. Health managers discussed the roles of the central Ministry of Health (MoH) and the DoH in restructuring.

Discussions were in English and Arabic. Comments were transcribed by three note takers, two of whom were bilingual Iraqis, to ensure that reflections from participants are fully captured and translated into valid recommendations, and that nothing was missed by misinterpretation and language barrier. Computerized notes were reviewed with key informants attending and edited for additional comments and clarifications. Names or positions of participants were not recorded in the data nor linked to particular statements or positions.

For comparisons of service availability following liberation from ISIS control, datasets from MoH/UNICEF 2017 national survey of health facilities in all districts in each of Iraq’s 19 governorates were made available for analysis.

Background information on health systems resilience in conflict was searched in peer-reviewed publications, internet references and references from humanitarian organizations.

## Results

In the analysis of the transcribed information common recurring themes were identified. In addition to the three components of resilience, themes such as community connectedness, health worker capacities, external health system support, previous strategy during conflict, and vulnerabilities were assessed.

*1. Absorption* The consensus of health managers was that the conflict caught health services in four governorates unprepared. The ISIS assaults on Erbil (Sept 2013) and seizure of Ramadi (Jan 2014) raised little initial concern among the other governorates at the time. Health facilities were caught by surprise when their area was enveloped by ISIS. The central MoH provided little guidance or support in preparation for conflict at clinics and hospitals. Many people, including health workers, fled the ISIS offensive. The initial response in most places was a struggle for continuity of basic services, with a reduced health work force and limited supplies. Roads were impassable in many places, blocking supplies and hospital referrals. The PHC facilities which did manage to absorb the initial shock while continuing some services did so largely as the result of work by individual health workers with support by community members. In this initial stage, pharmacies and private clinics played an important role as alternatives to the public sector. The Al Anbar team believed its coping capacity was stronger because of strategies developed during the earlier (2004–2007) Al Qaeda (AQI) occupation. Al Anbar may also have coped better because of the tribal cohesion developed during the Al Anbar awakening (2004–2009).

*2. Adaptation* The communities and health services developed various alternative approaches to continue providing care after the initial shock. The extent of adaptation varied greatly among locations. In most locations, MNCH preventive and promotive services at the PHC level stopped. Health facilities would adapt to meet primarily urgent situations such as injuries or communicable diseases. At some hospitals where health workers fled the threat of ISIS, health workers from outlying PHC clinics were moved to staff the hospitals that remained in government control. In Ninawa, the ISIS caliphate created a health directorate and fee-for-services in occupied areas. Hospitals, rather than PHC clinics were a priority for ISIS to care for their injured fighters. Residents hesitated visiting PHC clinics, fearing the religious police. Ill persons often consulted health workers in their houses at night for security reasons which built cohesion between health workers and the community.

In areas of the four governorates under government control, some NGOs or UN organizations were able to support health worker salaries to sustain services. Many health workers remaining in contested or insecure areas were not regularly paid. During ISIS occupation, salaries for government employees were deposited in adjacent governorates for eventual health worker payment, an adaptive measure by central government.

Overall, trust in the public sector health services deteriorated substantially as heath facilities were unable to adapt to meet community health needs. Private pharmacies managed to function in some areas and some even flourished by being able to acquire medicines through irregular supply routes. A few private clinics also functioned, run by public sector doctors working on the side, but many households could not afford private care.

In governorates partially occupied by ISIS, those areas under government control received large numbers of Internally Displaced Persons (IDPs). These governorates were struggling to provide services both for the IDPs and the resident population. Some international NGOs and UN agencies, especially UNICEF and WHO, were able to support PHC services for IDPs with MNCH supplies. This external support relieved stress on local health structures. During this time, many health workers as well as the population developed persisting anxiety and depression, with minimal if any mental health services available.

The MoH relocated the DoHs offices to Baghdad for Al Anbar and Sala al-Din, while for Ninewa, backup offices were established in Erbil and Dohouk (Kurdistan) to provide what remote management support was possible.

UN Agencies could, at times, negotiate with ISIS for some services, through facilitators and the tribal leaders, allowing the health teams to conduct vaccination outreach services. Initially ISIS permitted the vaccination services in the mosques and schools and later they accepted the house-to-house vaccination campaigns. During ISIS control of Ninewa, in person workshops to facilitate changing to bivalent oral polio (OPV) vaccination were not possible; however, ISIS did allow online training of health workers.

*3. Restructuring services* In many areas, health services restructuring was still ongoing in 2020. Over 1.4 million Iraqis remain displaced within Iraq [16]. Internally Displaced Persons face barriers that prevent their return home. These include others living in their house, disputed land ownership, presence of unexploded ordnance and lacking resources for house repairs owing to the head of household having been killed or being in prison. Some health care previously provided to IDPs stopped as international agencies closed programs, leaving IPDs in a precarious situation. Community members in many areas worked with health workers to carry out structural and clean-up actions needed, allowing non-functional PHC services to restart quickly, providing MNCH services such as nutrition and EPI services. UNICEF was active in supporting the MoH in the restoration of MNCH services.

Hospitals were heavily damaged in some places with the number of functional hospital beds in 2019 below pre-crisis level [17]. Other hospitals were functioning in temporary facilities. Many hospital specialists have left, though some were returning to work, while maintaining their residence elsewhere. In areas of Kirkuk, local ethnic or tribal conflicts continued, causing health workers who are not from these areas to decline postings here. Rebuilding the trust is proving difficult, as many persons felt let down by health services during the crisis. However, some facilities which previously enjoyed good community connections continued with strong community trust.

In 2017, the MoH with UNICEF support, assessed the status of health facilities from selected districts in all governorates of Iraq including those in areas newly reclaimed from ISIS. Assessments of availability, functionality, and provision of MNCH services are shown in Table 1. Data were compiled from different sources, including a nationally administered survey, national health information data and internal MoH/UNICEF survey data [18]. Some facilities in these four governorates may not have been under ISIS control at all, or only for a brief time, making the data not fully comparable across governorates.

**Table 1:** Status and services availability in primary healthcare facilities located in four conflicted-affected governorates compared with non-conflict areas within Iraq that offer MNCH services, 2017

	Anbar	Ninawa	Kirkuk	Salah al-Din	Non-conflict
Total facilities	N = 171	n = 186	n = 113	n = 86	n = 2699
Functioning*	137 (80.1%)	155 (83.3%)	86 (76.1%)	83 (96.5%)	2687 (96.2%)
Nonfunctioning	34 (19.9%)	31 (16.7%)	27 (23.9%)	3 (3.5%)	82 (3.8%)
*Proportion of facilities that offer Primary Health Care services*					
Immunization	102 (59.6%)	97 (52.2%)	76 (63.7%)	52 (60.5%)	1365 (63.7%)
Family planning services	22 (12.9%)	36 (19.4%)	45 (39.8%)	10 (11.6%)	688 (32.1%)
Antenatal care	44 (25.7%)	67 (36.0%)	63 (55.8%)	27 (31.4%)	1111 (51.8%)
Postnatal care	42 (24.6%)	55 (29.6%)	61 (54.0%)	26 (30.2%)	1000 (46.7%)
Basic lab testing	63 (36.8%)	64 (34.4%)	63 (55.8%)	38. (44.2%)	1147 (53.5%)
Blood typing	42 (24.6%)	50 (26.9%)	40 (35.4%)	27 (31.4%)	978 (45.6%)

UNICEF/MoH data analyzed with permission

*Functioning: regularly seeing patients during the normal operating hours for PHC clinics

### Differences among governorates

*Health facility functionality.* The table shows variable effects on functionality of health facilities (defined as seeing patients during regular clinic hours) in the main four ISI-affected governorates, ranging from 23% of total facilities being nonfunctional in Kirkuk at time of analysis, to only 3.5% in Salah al-Din. In some governorates, not all facilities were in ISIS controlled areas. Even among functional PHC facilities, availability of essentials MNCH services were variable. The table shows that family planning, antenatal care and postnatal care were less available compared with immunization services which are better sustained. Also, low availability of basic laboratory tests (mostly on range of less than 50%), including blood typing, represented a consistent feature across the four governorates.

*Resilience themes.* There were recurring themes experienced across all four governorates such as health workers who felt isolated from central government assistance, leaving them ill prepared to absorb the impact of conflict. Few facilities had emergency plans and most saw community trust in health services ebb as the care they could provide was increasingly limited. There were also differences among governorates which determined adaptive and restructuring approaches. To some extent this could depend on the extent of the governorate remaining under government control (Fig. 1). Some areas believed they had greater capacity to develop adaptive measures from previous experiences of the 2003 invasion and the succeeding waves of conflict. Participants reported tribal tensions negatively affected Kirkuk particularly, whereas tribal traditions were an adaptive strength in parts of Al Anbar, perhaps stemming from the 2006 “awakening” movement. In Ninewa governorate it was reported that Mosul’s sophisticated health facilities adapted to a much lower functional level after ISIS invasion, yet the restructuring continues to prove difficult due to major infrastructural conflict damage.

From some governorates, large proportions of the population fled to neighboring governorates, straining services in these host areas. This was noted by participants from Al Anbar and Kirkuk where many were displaced within government-controlled areas, placing a heavy demand on functional services for these governorates, especially as the health status of the displaced was often poor. In places within Kirkuk and Salah al-Din, international organizations were able to ease some of the demands on the directorate of health, with the World Health Organization and UNICEF particularly active. Differing levels of compensation of health staff for salaries lost during conflict years has resulted in a limited numbers of health workers willing to work in some governorates. In all four governorates, participants reported mental health and psychosocial problems as well as physical injuries among health workers, though these varied with the local context. These were frequently cited as reasons for limited availability of health workers to help restructure services. In some areas, ISIS deliberately targeted health workers, leaving lasting trauma and a hesitancy to return to previous locations [5].

## Discussion

This assessment of health systems resilience in the four governorates of Iraq controlled by ISIS from 2014 to 2017, found a devastating impact on health services. In the face of the initial shock, health facilities had limited capacity to continue providing services. Adaptation to functioning under ISIS did not fare much better, largely cut off from support and medical supplies from the MoH. Local initiatives such as sick community members consulting health workers in their homes at night were a creative adaptation. Some facilities in Al Anbar had learned adaptation workarounds from earlier incursions by Al Qaeda. These included bringing in supplies by obscure or rarely used routes and health workers carrying medical supplies on foot through ISIS areas at night from safe government-controlled areas using local knowledge. UNICEF-supplied vaccines were able to reach some ISIS controlled areas at times, arranged through unofficial negotiations, a transformative stage activity. The transformative process to function effectively in the post-conflict environment is not yet a completed process in many locations, limited by insufficient funds, and a lack of human resources. Support by UNICEF has hastened restructuring of MNCH care as part of PHC services in some locations, particularly through construction of vaccine distribution points in badly affected areas.

Despite a 40-year history of almost continuous conflict, Iraq had not developed robust emergency management plans either at a facility or DoH level. At the central MoH level, disaster preparedness was not a priority. The massive disruption following the 2003 invasion and the extensive displacement from the 2005–2007 civil war took a heavy toll on Iraq’s health system. The migration of many doctors diminished leadership and management continuity. Although the MoH had earlier announced a reorientation toward primary health care, the public sector has retained a centralized hospital-oriented function, flirting only briefly with concepts of decentralization [4]. Building governorate level disaster response capacity was deemed important by health managers from the four areas, yet it would be difficult to implement this without support from local authorities. Despite the lack of preparedness, the MoH did create adaptive strategies such as depositing the pay for health workers under ISIS control in adjacent governorates, should access become periodically available.

The initial shock of seizure by ISIS fell most heavily on the PHC clinics (PHCC), as seen in the table. Hospitals falling under ISIS control tended to remain more functional, as they were expected to care for injured ISIS fighters. Many health workers fled, effectively halting MNCH services. Supply chains were interrupted. Some people hesitated to attend the remaining functional PHCCs as they doubted the medicines they might need would be present, or feared encounters with *Al Hesba*, the religious police [19]. Where services were able to adapt, there was usually a strong cohesion between communities and health facilities and committed health staff. Some patient consultations took place in the evenings at the homes of health workers, where people felt safer. In places, private clinics and pharmacies could meet some basic needs when the public sector could not. In Iraq, the pharmaceutical sector is largely private with its own supply networks giving it added flexibility, even in conflict-affected areas. Private clinics are generally operated by public sector doctors in off hours. Their presence in the conflicted areas suggests an inability of the public sector to fully meet a population’s health needs, as well as the capacity for discretionary spending.

In discussions with health care managers from the four governorates there were common recurring conflict-resilience themes which form the following six recommendations:

1. *Building community cohesion*. Where community support was present, health facilities and health systems functioned better during shock and adaptation. The involvement of religious and tribal leaders helped to open a humanitarian corridor, access negotiation, protect health facilities and could sustain them through access to community power sources and provide warning of potential threats. Community cohesion could start with closer involvement of local councils in health facility support, especially at the PHCC level. A recurring theme was the need to restore community trust which was lost from the collapse of health services during insecure times.
2. *Developing disaster plans.* Health managers felt local insecurity threats persist and planning for seemingly inevitable future shocks was urgently needed. This should start with convening health system leadership to share experiences and successful strategies as a basis of disaster planning. Plans should be developed at different levels but with community participation, and health staff helping develop community emergency plans. Plans for ensuring continuity of care for people in the community with special needs should be made. Training is needed for health leaders and workforce to respond and adapt effectively in emergencies. Participants felt that the DoH could help functionality of facilities and build emergency capacities and decision-making skills among health workers.
3. *Developing DoH (governorate) based disaster strategies.* Creation of an Emergency Operations Center (EOC) at governorate level could coordinate a disaster response. Early warnings could be provided, and additional human resources and supplies deployed for a rapid response. The need for effective communications between different levels was seen during the conflict time. Increasing buffer or reserve stocks of medicines and supplies would help sustain services following initial shocks. Reserve funding to sustain services in crises could help meet emergency needs. Health managers felt that the central MoH should take the responsibility to coordinate disaster management planning at different levels.
4. *Cross-sector coordination.* Development of DoH emergency management capacities would link other sectors at the governorate and district level for a rapid response to disaster needs. Resilience of the health sector depends on support from other elements in the public sector, and during the crisis years, these links need to be in place. Further to these recommendations, there are potential public–private options for transportation, pharmaceuticals and human resources as well as access to private care sources which could be very important in crises situations [20].
5. *Rebuilding the heavily damaged health sector.* Health managers saw fully restoring health facilities as in rebuilding trust in the health sector. Both ISIS control and the military action at liberation seriously damaged the health sector making it unable to meet many basic health service. Even several years after the elimination of ISIS, many health managers did not see a comprehensive infrastructure rebuilding strategy in place. Resources for health infrastructure restructuring so far have largely come from individuals, communities, or international organizations but little from central government.
6. *Building Capacity of Care Providers*. Erbil workshop participants felt health facility staff needed new skills to plan, respond and adapt to crisis, a need that was mentioned frequently. Particularly, the need to develop plans for continuity of care during crises was identified. The need for ways of building psychological resilience among health workers was seen as especially important given the emotional trauma experienced by many health workers during the ISIS occupation. Although this topic was discussed in the context of renewed conflict, such skills would be useful in other crises such as disease outbreaks, population displacement by natural disasters and the COVID-19 pandemic.

### Approaches to building resilience

There is an expanding interest in approaches to managing resilience in health services from the governance as well as the health systems perspectives. Although there are many steps that can be taken at the health facility and local levels, resilience of systems is linked to strong management processes which contribute to robust governance at the systems level [21, 22]. This requires a systems thinking approach to creating a resilient health service as there is a high probability of continuing risk and stress in Iraq. Among those approaches particularly relevant to resilience of the Iraq health sector are participation, deliberation, knowledge, and management of uncertainty. In their discussion, health managers from the conflict-affected areas recognized that resilience is affected by interdependent systems interacting with each other to influence the response to shocks at various levels of the health system. At the governorate level, decentralization of health service authority could facilitate development of DoH emergency operations crisis management and could facilitate building skills in areas such as deliberation, discussion, and management of uncertainty. Health managers believed that the central MoH in Baghdad had not supplied the guidance or support they felt necessary to build governorate and facility level to manage uncertainty. Initial efforts at decentralization in Iraq were set out in 2014 but were subsequently put on hold [23, 24]. Sharing successful strategies among the four conflict-affected governorates could identify common vulnerabilities and successful approaches. Health managers participating in this current study were unanimous in believing that similar events to the ISIS assault would happen again. They were equally fearful that the health system would be no better prepared when the next crisis appeared. Unfortunately, the time is slipping away to fully document health service resilience and document lessons learned from the ISIS events.

At the other end of the health system, participants repeatedly identified participatory engagement with community leadership as critical for developing trust, a key ingredient in health system resilience. Community-based care was shown to be an essential component for resilient health systems during the West African Ebola outbreak [25]. There have been a number of models of successful community-health facility processes [26, 27]. The Health Visitors program, which exists in southern Iraq, could build such engagement on a wider scale if the program is expanded to other parts of Iraq [4]. This program has built strong engagement between communities and PHC clinics, building health awareness and mapping community vulnerabilities [28]. Community care programs can be an important component in downward health system accountability and support participatory engagement.

### Limitations

This report is based on separate focus group discussions with DoH health managers from each of the four ISIS-affected governorates and key informants. Thus, the focus is on resilience as seen mostly from governorate level, although many managers had served in health facilities during the ISIS years. There were no representatives from affected communities to give their views. Representatives from the central MoH attended and reflected views from Baghdad. Although all parts of Iraq have been affected by conflict in the past 40 years, we concentrated on these four governorates where experiences are still fresh in the mind of managers who lived through ISIS conflict times. The MoH/UNICEF health facility survey data cited may not have fully captured information from facilities under ISIS control.

## Conclusion

The 2014 ISIS invasion of four Iraqi governorates revealed health services with little crisis preparation and limited absorptive or adaptive capacities. Health services struggled to maintain essential PHC services during crisis years, although private services could sometimes supplement the public sector. Many facilities were reduced to supplying services only for acute illnesses and injuries. The health system transformative phase following the ISIS defeat seems stalled, although most of invaded areas were liberated in 2017. Building a more resilient system at local governorate level, with capacity to better withstand different forms of stress and shocks need to be prioritized in health planning. Health managers from the four governorates felt that experiences during the ISIS years should be consolidated as a basis for crisis planning at the facility and governorate levels before they were lost from organizational memory.

## Data Availability

The health facility dataset cited is available on request from shibrahim@unicef.org.
